# Diagnosis of tuberculosis: the experience at a specialized diagnostic laboratory

**DOI:** 10.1186/1477-5751-10-16

**Published:** 2011-11-18

**Authors:** Anita Mashta, Pooja Mishra, Sonia Philipose, S Tamilzhalagan, Hanif Mahmud, Sangeeta Bhaskar, Pramod Upadhyay

**Affiliations:** 1 Product Development Cell, National Institute of Immunology, Aruna Asaf Ali Marg, New Delhi 110067, India; 2 New Delhi Tuberculosis Center, JLN Marg, Delhi Gate, Delhi 110002, India

## Abstract

This work describes the experience at a tuberculosis clinical laboratory where relatively new TB diagnosis technologies; nucleic acid detection of two target strands, IS*6110 *and *devR*, by PCR and microscopic observation drug susceptibility (MODS) were used. The LJ culture was the gold standard. This evaluation was done from August 2007 to July 2009 on 463 sputum samples of tuberculosis suspects at a specialized tuberculosis clinic in Delhi, India.

None of the tests we evaluated can accurately detect the presence or absence of *Mycobacterium tuberculosis *in all the samples and smear microscopy was found to be the most reliable assay in this study.

The PCR assay could detect down to 2 pg of H37Rv DNA. Sensitivity, specificity was 0.40, 0.60 and 0.19, 0.81 for smear positive (n = 228) and negative samples (n = 235) respectively. In the MODS assay, sensitivity, specificity of 0.48, 0.52 and 0.38, 0.76 was observed for smear positive and negative samples. Sputum smear microscopy had sensitivity of 0.77 and specificity of 0.70.

## Introduction

Despite the availability of effective and inexpensive therapy, tuberculosis (TB) is one of the leading causes of death from an infectious disease. It is believed that the clinical management of TB is made more difficult by the lack of a simple and effective diagnostic test. Correct and timely diagnosis of TB is very important to achieve higher compliance with the treatment, reduce transmission and to reduce the development of drug resistance.

Along with the emergence of evidence based diagnosis approaches [1], a number of new technologies have been introduced [2]. These include light-emitting diode (LED)-based fluorescence microscopy [3], automated liquid culture systems such as BacT/ALERT MP [4], interferon-gamma release assays [5], etc.

Recently published meta-analysis and reviews make us to believe that the future of TB diagnosis is bright. On the other hand, WHO and other organizations such as FIND (Geneva) perpetually call proposals for the development of simple and cost effective tests for TB diagnosis. This suggests that the current scenario is far from satisfactory and not all the claims made by the researchers and companies regarding the sensitivity and specificity etc. of the TB diagnostic tests are valid in actual 'field conditions'.

We carried out an evaluation of relatively new TB diagnosis technologies. In addition to the direct sputum microscopy, we performed nucleic acid detection of two target strands, IS*6110 *and *devR*, by PCR and MODS. LJ culture was used as the gold standard.

The direct sputum microscopy is still the primary means for diagnosis of TB in India. Nucleic acid amplification tests (NAATs) in principle have high sensitivity and specificity. Due to the limited utility of *IS*6110 for TB diagnosis in North India [6], we included another target gene sequence, *devR*, which is expressed during hypoxia conditions [7].

The existence of a toxic glycolipid, trehalose 6-6' dimycolate (cord factor) of *M. tuberculosis *was known from a long time [8-10]. Darzins and Fahr [11] demonstrated the difference between pathogenic strains and non-pathogenic strains on the basis of cord forming properties of the mycobacterium. The cording of *M. tuberculosis *on agar and its diagnostic potential was later demonstrated by Lorian in 1966 [12,13]. It more recent time, the ability of virulent *M. tuberculosis *to grow and from cords has been demonstrated by a few groups [14,15]. Both of these groups reported very high sensitivity of the test. A validation of such a test was carried out in Peru [16] and given a new name, microscopic observation drug susceptibility (MODS) to this assay.

This work describes the experience at the specialized tuberculosis clinical laboratory. We observed disturbing inconsistencies in results and it is hard to find explanations for the same.

## Results

### The detection limits of PCR

Serial dilutions of 200 ng H37Rv DNA were made in six steps such that the amount of DNA in the final dilution was 2 pg. PCR was performed on these dilutions for the IS*6110 *gene and the lower detection limit of IS*6110 *sequence by PCR was found to be around 2 pg.

### Typical results obtained from clinical samples

**Figure 1:**
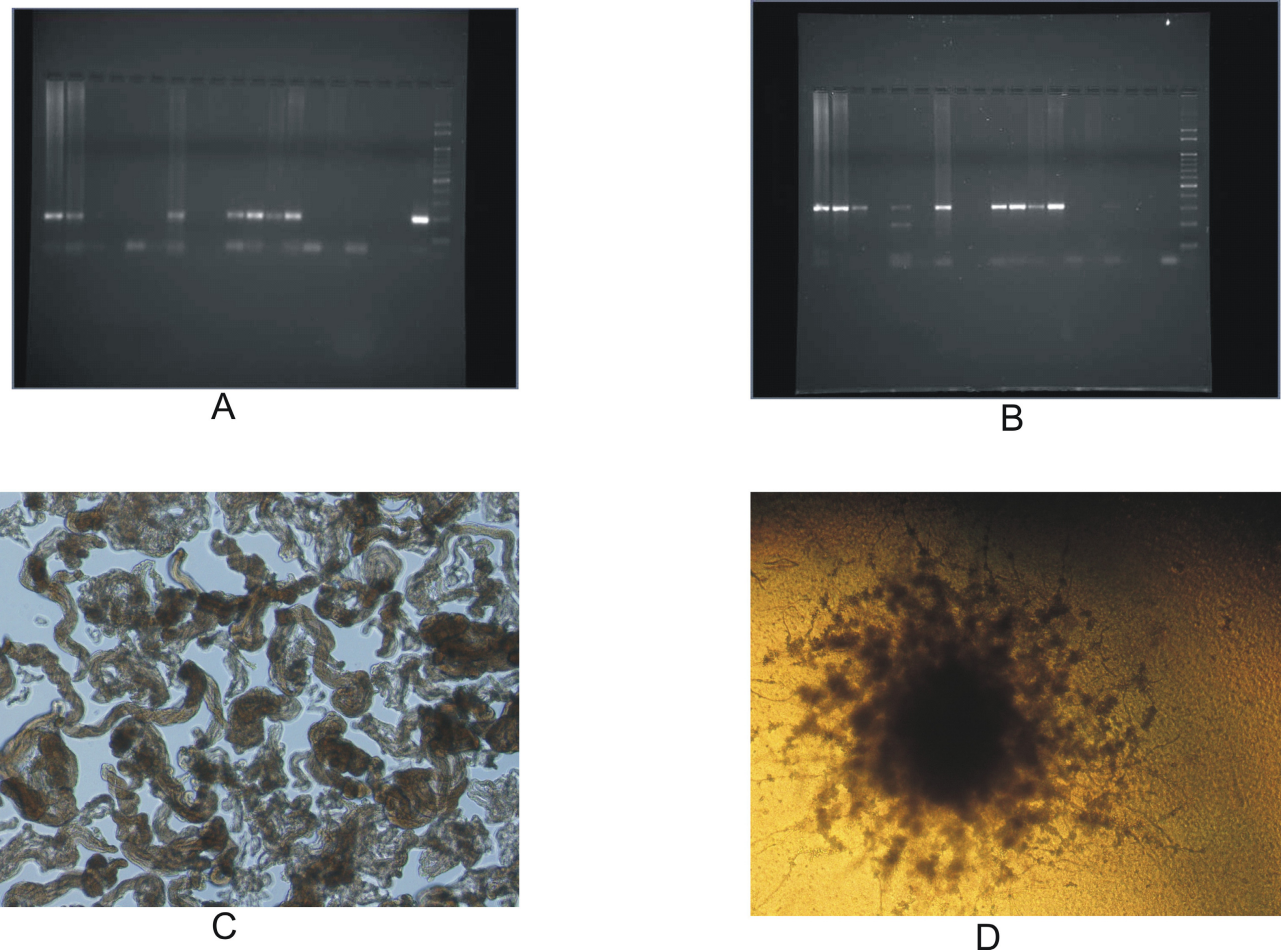
**Some representative results of the study**. A typical gel picture showing amplification of IS*6110 *target sequence (A) and *devR *sequence (B), a typical formation of cords by H37Rv (C) and sample (D) in the MODS assay.

Some of the typical results obtained from clinical samples are shown in Figure 1. The distinct 197 base pairs and 308 base pairs amplification bands of IS*6110 *and *devR *respectively are neatly visible in DNA isolated from sputum samples.

Results obtained by different diagnostic tests, over all (463) as well as divided between smear positive (228) and negative (235), are compared against the culture reports. These are discussed below and compiled in Table 1, 2 and 3.

**Table 1:** Summary of data for PCR and LJ culture.

PCR and LJ culture									
	Smear +ve			Smear -ve			Overall		
	Culture +ve	Culture -ve	Total	Culture +ve	Culture -ve	Total	Culture +ve	Culture -ve	Total
PCR +ve	60(26.3)	32(14.0)	92(40.4)	9(3.8)	35(14.9)	44(18.7)	69(14.9)	71(15.3)	140(30.2)
PCR -ve	88(38.6)	48(21.1)	136(59.6)	39(16.6)	152(64.7)	191(81.3)	123(26.6)	200(43.2)	323(69.8)
Total	148(64.9)	80(35.1)	228(100)	48(20.4)	187(79.6)	235(100)	192(41.4)	271(58.5)	463(100)
**Analysis of data**									
	value	95% Confidence Interval		value	95% Confidence Interval		value	95% Confidence Interval	
Sensitivity	0.40	0.32 to 0.49		0.19	0.089 to 0.32		0.36	0.29 to 0.43	
Specificity	0.60	0.48 to 0.71		0.81	0.75 to 0.86		0.74	0.68 to 0.79	
PPV	0.65	0.55 to 0.75		0.20	0.098 to 0.35		0.49	0.41 to 0.58	
NPV	0.35	0.27 to 0.44		0.79	0.73 to 0.85		0.62	0.56 to 0.67	
Likelihood Ratio	1.01			1.00			1.37		

The comparison was carried out individually on smear +ve and -ve samples. Indicated values are n(%).

**Table 2:** Summary of data for MODS and LJ culture.

MODS and LJ culture									
	Smear +ve			Smear -ve			Overall		
	Culture +ve	Culture -ve	Total	Culture +ve	Culture -ve	Total	Culture +ve	Culture -ve	Total
MODS +ve	72(31.6)	38(16.7)	110(48.2)	17(7.2)	45(19.1)	62(26.4)	89(19.2)	83(17.9)	172(37.1)
MODS -ve	76(33.3)	42(18.4)	118(51.7)	27(11.5)	146(62.1)	173(73.6)	103(22.2)	188(40.6)	291(62.8)
Total	148(64.9)	80(35.1)	228(100)	44(18.7)	191(81.3)	235(100)	192(41.4)	271(58.5)	463(100)
**Analysis of data**									
	Value	95% Confidence Interval		Value	95% Confidence Interval		Value	95% Confidence Interval	
Sensitivity	0.49	0.40 to 0.57		0.39	0.24 to 0.55		0.46	0.39 to 0.54	
Specificity	0.52	0.41 to 0.64		0.76	0.70 to 0.82		0.69	0.64 to 0.75	
PPV	0.65	0.56 to 0.74		0.27	0.17 to 0.40		0.52	0.44 to 0.59	
NPV	0.36	0.27 to 0.45		0.84	0.78 to 0.89		0.65	0.59 to 0.70	
Likelihood Ratio	1.02			1.64				1.51	

The comparison was carried out individually on smear +ve and -ve samples. Indicated values are n(%).

**Table 3:** Summary of data for microscopy and LJ culture.

Culture and Smear Microscopy			
	Culture +ve	Culture -ve	Total
Smear +ve	148(32.0)	80(17.2)	228(49.2)
Smear -ve	44(9.5)	191(41.2)	235(50.7)
Total	192(41.5)	271(58.5)	463(100)
**Analysis of data**			
	Value	95% Confidence Interval	
Sensitivity	0.77	0.70 to 0.83	
Specificity	0.70	0.65 to 0.76	
PPV	0.65	0.58 to 0.71	
NPV	0.81	0.76 to 0.86	
Likelihood Ratio	2.61		

Indicated values are n(%).

### PCR assay

The summary of data and analysis is shown in Table 1. For the smear positive samples very low sensitivity 0.40 was observed which deteriorated to 0.19 for smear negative samples. The likelihood ratio of 1.37 for overall PCR samples suggests that it could be of some importance to finally classify a sample.

### MODS assay

Results and analysis are shown in Table 2. The sensitivity figures observed with smear positive and negative samples were 0.48 and 0.38 respectively; which make MODS results similar to PCR. Although slightly better likelihood ratio of 1.53 for MODS may makes it more preferred over PCR assay.

### Sputum smear microscopy

Results and analysis are summarized in Table 3. The sensitivity and specificity for smear microscopy was 0.77 and 0.70 respectively. The higher likelihood ratio of 2.6 can significantly influence the final outcome of the readout.

## Discussion

### Limitations of the study

It is essential to highlight the limitations of this study before any meaningful conclusion can be drawn.

1. We had access to only the results of the diagnostic assays and therefore it is not possible to classify specimens on the basis of case history, age, sex etc.

2. We have used LJ culture as the gold standard and this has led to some degree of underestimation of test accuracy as some of the liquid culture assay like BACTEC, MGIT etc. have around 10% higher sensitivity [17]. When the sensitivity of gold standard (LJ culture in our case) is not 100% and it is rarely non specific; the sensitivity and not the specificity is the important parameter when comparison between LJ culture and 'new tests' are made.

3. Our procedure for the MODS assay was similar to the resources provided on http://www.modsperu.org/, but there were minor differences.

We have used two well established sequences for the NAATs [18,19] and we took extraordinary measures to remove PCR inhibitors. The observed inconsistencies in NAATs are perhaps a confirmation of the observation that 'in-house' NAATs produce highly inconsistent results and have lower and highly variable sensitivity in smear negative specimens [20,21].

The MODS is a very interesting liquid culture based diagnostic assay [22]. Very high sensitivity of detection, 97.8% or similar [23] has been reported for this assay. We were surprised that how in our case the sensitivity of MODS is so different.

The reason perhaps is the flawed generalization that all pathogenic *Mycobacterium tuberculosis *form cords. Mycolic acids and mycolyl glycolipids are unique and ubiquitous components of mycobacterial cell envelopes. Among such components, TDM was first isolated as cord factor from highly virulent *Mycobacterium tuberculosis *showing cord-like growth on the surface culture in liquid media. Later it was demonstrated that most species of culture-able *Mycobacteria *including the BCG has TDM on their surface [24]. Paradoxically, most tissue damage in TB disease is not caused by *Mycobacterium *itself; instead it is caused by body's response towards the *Mycobacterium *[25]. Therefore a generalization of the virulence of *Mycobacterium *on the basis of its surface glycolipid or the property to form cords cannot be accurate.

We did not have access to the profile and the case history, such as for how long they were on antibiotics treatment etc. of all the patients and therefore it is not possible to provide an explanation for lower specificity of microscopy. In a realistic situation, not every patient has or shares his/her case history with the hospital and such samples are often excluded from most studies. After such exclusions, we cannot hope to see the overview of the clinic because such patients also get treatment on the basis of their test reports. We therefore decided to include all the samples even if they come without the 'case history' and we could see a scenario which is the 'true' reflection, though it is difficult to interpret.

Possibly, the reason of poor correlation among different tests is due to the fact that the limits of errors of different methods and uncertainties of samples vary dramatically from a research laboratory to a clinical laboratory. In a clinical laboratory, the diversity of samples and limits of errors are generally high. In this study, we observed the amplification of errors and limitations when different methods (including the 'gold standard') were put together. Although the sensitive assays like NAATS, LJ and MODS can detect fewer *Mycobacterium *but with every addition of steps in the methodology we introduce additional errors and uncertainties also; intricate steps are likely to add more errors.

Overall, our data suggest that sputum smear microscopy is a little better than any of the tests we evaluated. It is the cheapest, simplest and the most straightforward assay for TB diagnosis.

## Materials and methods

All the steps were taken to comply with the Standards for the Reporting of Diagnostic accuracy studies **(**STARD) checklist http://www.stard-statement.org/.

### Ethics Statement

National Institute of Immunology (NII) only received anonymous, coded sputum specimens with no patient identifiers and it was approved by the Institutional Human Ethics Committees of the NII, project serial number IHEC#21/05.

### Recruitment and Specimen collection

NDTB center fetches samples from a large geographical area of North India. NDTB center is a Central TB Division Ministry of Health, Government of India, accredited laboratory and training center. Necessary details for the accreditation are given on http://www.tbcindia.org/documents.asp.

**Figure 2:**
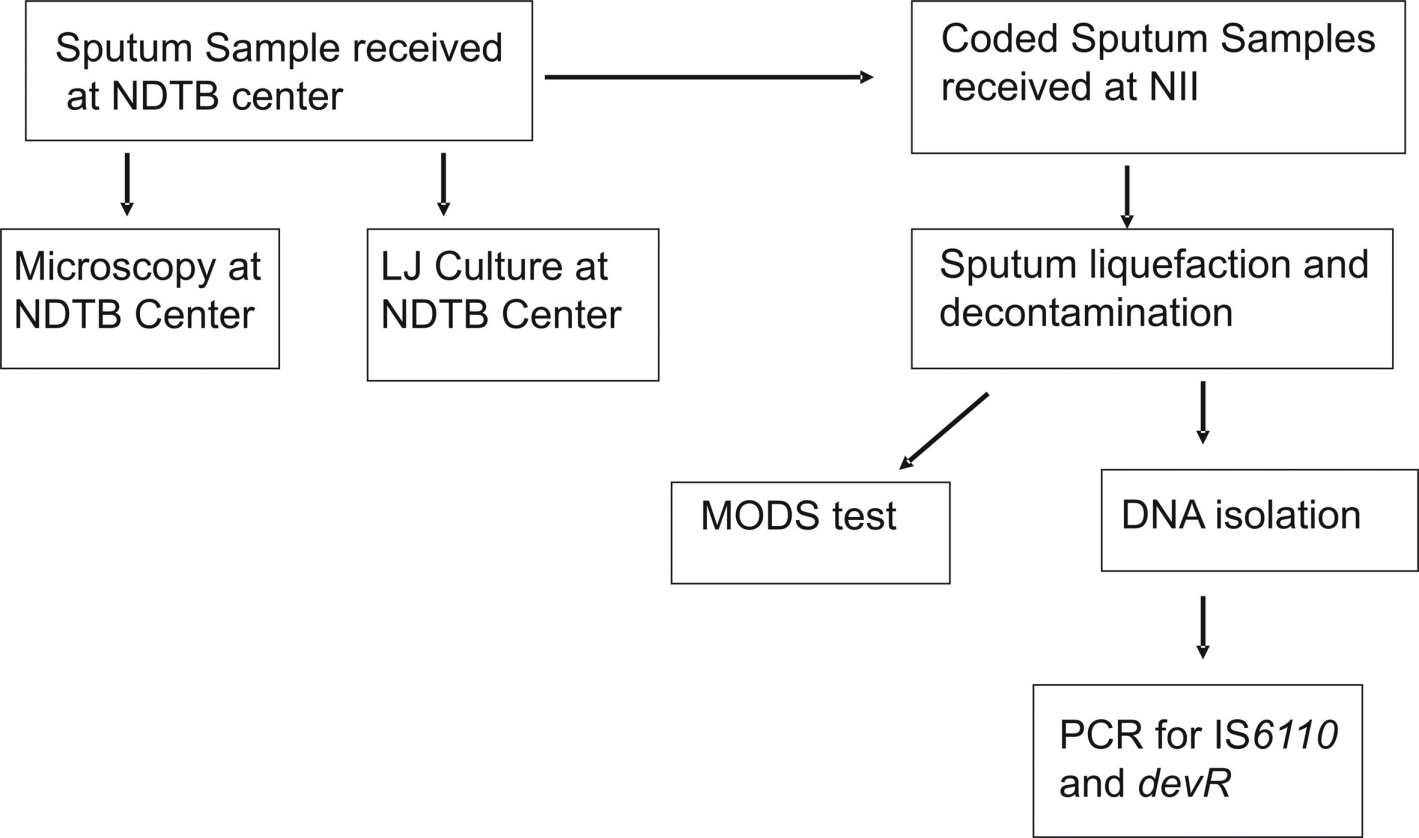
**Flow chart describing movement and processing of sputum samples**.

Many (50-100) sputum samples of TB suspects are received at the NDTB center daily and microscopy and LJ culture are performed the same day. NII received coded, single sputum sample from each patient from August 2007 to July 2009 on a working day, either in the second or third week of the month. Due to the non-availability of reagents, etc. samples could not be collected every month. All the samples received in the NDTB laboratory on that particular day were included in the study. Flow chart shown in Figure 2 describes the movement of samples.

On a collection day, samples were divided in two aliquots and one of the aliquots was transported to NII on the same day on ice packs in double seal, air tight containers. Transportation time was than 1 hour. PCR and MODS assay were performed within 12 hours. All the tests were performed by highly skilled technicians and trained research fellows. All of them had undergone mandatory training of two months for handling Mycobacterium infected samples and setting up MODS and PCR tests.

### Blinding

HM at NDTB center was having the codes of samples. At NII, PU coded the samples again and ensured that readout of all the assays remain blinded to each other. All the codes were open only after completing the study. None of the details except smear microscopy result and culture report of specimens were revealed by the NDTB center.

### Microscopy

Detailed procedure used for microscopy is given at http://www.tbcindia.org/documents.asp. Briefly, sample was placed in the centre of the slide, air dried for 15-20 minutes and fixed by passing through a flame. Filtered carbol fuchsin was poured to cover the entire slide and left aside for 5 minutes. Free carbol fuchsin stain was then washed off under running water. The slide was decolorized by 25% sulfuric acid treatment for 2-4 minutes and counterstained by 0.1% methylene blue for 30 seconds. The slide was washed under running water, dried and around 100 fields were examined under the microscope. Microscopy was done by experienced technicians. They had undergone in house training for two weeks at the beginning of their carrier. All technicians annually under go RNTCP training for 10 working days. None of the technician at NDTB center has less than 5 years of experience. For the purpose of this study, samples were classified either as positive or negative without any gradation of smear positive samples.

### LJ culture

Samples were liquefied by 4% NaOH solution for 20 minutes, centrifuged at 3000 g and pellet was washed twice with distilled water. One loopful of concentrated pallet was inculated on to the LJ slope prepared in McCartney bottle. Growth of *Mycobacterium *was examined every week. Contaminated cultures were identified within a week's time. Such cases were less than 4%. These were not excluded from the study and patients were called again to collect another sample. Cultures were incubated for eight weeks before classifying them as negative. Plates were examined till 8 weeks before considering them as negative. NIACIN production, catalase activity at 68°C and nitrate reduction tests were performed to ensure that NTMs were not counted as culture positive. Detailed procedure is given at http://www.tbcindia.org/documents.asp.

### MODS assay

#### Isolation of cells

A thorough standardization of NaOH concentration in sputum liquefaction solution and duration and force of centrifugation was conducted. Mucus in the sputum sample was liquefied by mixing 5 ml (maximum) sputum with equal volume of 1%NaOH, 0.5% N-Acetyl-L-Cysteine and 1.44% Sodium Citrate solution. After incubation at room temperature for 15 minutes this mix was centrifuged at 2000 g for 30 minutes. The resulting pellet was washed with PBS and re-suspended 1 ml PBS.

#### Setting up the assay

We followed the procedure outlined in http://modsperu.org/ with minor variations. Briefly, Middlebrook 7H9 broth medium with 10% OADC supplement and antibiotic mixture (Carbenicillin disodium salt-50 mg/l, Cycloheximide-0.4 mg/l, Amphotericin B-15 mg/l, Polymyxin B-Sulphate-26 mg/l and Vancomycin-10 mg/l) was taken in 24 well plate. All of these reagents were purchased from Hi-Media, India. Each well had 1.5 ml of the medium. Three different volumes, 10 μl, 20 μl, and 50 μl of cells isolated from sputum were inoculated in triplicates. Only one specimen was plated on a plate. Every plate had H37Rv culture and blank in duplicates as positive and negative control respectively. The culture plates were sealed from the all four sides and kept inside a polythene envelope and sealed again and incubated at 37°C. After 3 days cultures were examined daily to detect the formation of cords under an inverted microscope at 10× objective. Formation of cords in any of the triplicates, at any of plating concentration was classified as positive. Upon classifying a plate as positive, it was removed from the incubator and appropriately discarded. Specimens were incubated for two weeks before classifying them as negative.

### Nucleic acid detection

#### DNA extraction

After setting up the MODS assay, in the remaining fraction of cell suspension inhibitor removal solution containing 5 M GITC, 25 mM EDTA, Sarcosyl 0.5% w/v, 0.2 M β-mercaptoethanol in 50 mM Tris-Cl (Trisma base) pH 7.5 was added for removing PCR inhibitors for 15 minutes and it was washed with 50 mM PBS. DNA was isolated by spin column (MDI Miniprep kit) and suggested protocol was followed. Briefly, the pellet was re-suspended with 350 μl of BT-1 (MDI Miniprep kit) solution. 20 μl of 10% lysozyme (Sigma) was added to lyse the cell. It was incubated for 1 hour at 37°C. Then 5 μl of 0.1% Proteinase K (Bio Basic Inc.) and 1 μl of 10% RNase (Bio Basic Inc.) was added and incubated at 50°C for 30 minutes. 350 μl of BT-2 (MDI Miniprep kit) solution was then added and kept at 50°C for 30 minutes. It was then centrifuged at 2000 g for 2 minutes. The supernatant was transferred into spin column and centrifuged at 16,000 g for 2 minutes. The column was then washed with wash buffer and kept at room temperature for 15-20 minutes to evaporate the wash buffer. DNA was collected by placing 100 μl of MillQ water over the column and collected DNA was stored at -20°C. In every DNA extraction cycle, a specimen containing H37Rv culture and blank were included as positive and negative control respectively.

#### Preparation of test genomic DNA

H37Rv culture was grown in 7H9 medium with 10% OADC supplement. DNA was isolated by spin column (MDI Miniprep kit) and the yield was estimated by measuring the absorbance at 260 nm and 280 nm.

#### PCR assay

We used no 'industry standard' tuberculosis diagnosis PCR assay kit and all necessary standardization was 'in-house' and used extensively studied target sequences, *IS*6110 and *devR *for the PCR assay. iNtron Biotechnology kit was used to perform the PCR. A single PCR of 20 μl consists of 2 μl of 10X PCR buffer, 50 μM dNTPs, 0.2 μM of forward primer and reverse primer each, 0.75 Unit of Taq DNA Polymerase along with 2 μl test DNA solution and water. After an initial denaturation at 94°C for 5 minutes, 45 cycles of 94°C for 45 s (denaturation), 60°C for 45 s (annealing), 72°C for 45 s (extension) were performed on Eppendorf Mastercycler. After completing thermal cycles the final extension at 72°C for 7 minutes was carried out. Amplified amplicons were resolved in 2% agarose gel. In every PCR assay, confirmed genomic DNA of H37Rv and a blank were included as positive and negative control.

#### Primers and probes

*devR *gene

Amplicon length - 308 base pairs

Forward Primer - **177 **5'TGGCAACGGCATTGAACTGT 3' **196**

Reverse Primer - **484 **5'TAAGCAGGCCCAGTAGCGT 3' **466**

IS*6110 *gene

Amplicon length -197 base pairs

Forward Primer - **502 **5'TTCGGACCACCAGCACCTAACC 3' **523**

Reverse Primer - **698 **5' CCTTCTTGTTGGCGGGTCCAG 3' **678**

### Data analysis

The statistical analysis was performed using Graph Pad Instat software (GraphPad Software Inc.) version 3.05.

## Competing interests

The authors declare that they have no competing interests.

## Authors' contributions

AM, PM, SP and ST performed the PCR and MODS assay. HM supervised sample collection and was responsible for microscopy and LJ culture. SB analyzed the data. PU performed and was responsible for the PCR and MODS assay, analyzed data and wrote the paper. All authors read and approved the final manuscript.
